# Iodoform and Thyroidism

**Published:** 1910-06

**Authors:** A. Rendle Short

**Affiliations:** Demonstrator of Physiology, University of Bristol; Hon. Surgical Registrar, Bristol Royal Infirmary.


### IODOFORM AND THYROIDISM.

BY

A. Rendle Short, M.D., B.S., B.Sc., F.R.C.S.,

*Demonstrator of Physiology, University of Bristol;*
*Hon. Surgical Registrar, Bristol Royal Infirmary.*

---

The conclusions which I hope to establish are these:

1. That iodoform, when absorbed by the system, either from a wound or from the bowel, is excreted into the blood by the thyroid gland as iodothyrin, which is the active principle of the colloid of that gland.

2. That an excessive quantity will produce symptoms of acute thyroid intoxication.

Both the above appear to be thoroughly accepted by the modern school of physiologists and pharmacologists. I hope to add a third conclusion, which as far as I am aware has not previously been recorded.

3. That in the susceptible person iodoform may precipitate an attack of *chronic* thyroid intoxication, namely exophthalmic goitre.

It was demonstrated so long ago as 1896, by Baumann, of Freiburg, that the colloid of the thyroid gland contained far more iodine than any other organ in the body. This has been abundantly confirmed by every subsequent observer. The amount varies a good deal in different species. In herbivores it is abundant; in carnivores it is very scanty. This is probably a question of diet. Most vegetables contain iodine, but in the animal body it is present only in the thyroid (with the exception of occasional mere traces in the blood). In young children it is scanty, in some cases falling below our none too accurate means of estimation.

*The iodine is an essential constituent of the active principle of the colloid.* It has been proved, both by Roos and by Hunt and Seidell[3] (working in the U.S.A. Public Health Laboratory), that the activity of the colloid varies with the percentage of iodine. The relationship is so definite that leading manufacturing houses now standardise their thyroid extracts for therapeutic purposes by the iodine content, which should not fall below 0.2 per cent. of organic iodine.

*Deprivation of iodine is a cause, probably the only cause, of parenchymatous goitre.* It has been shown in North America that in some districts remote from the sea the vegetation is unusually deficient in iodine, and there was very serious commercial loss amongst sheep farmers by reason of the number of cretin lambs. Many of the sheep and dogs were goitrous. The introduction of iodiferous salt was completely successful in preventing this. Previously a pure rock salt had been used.[4]

I suggest that the success of Chalmers Watson and of Edmunds in producing goitre in fowls by limiting them to a meat diet was the result of iodine starvation.

The causation of human goitre has long been a problem of great interest. We know that it is in some way related to the drinking water. Goitre follows particular geological formations: in England the carboniferous limestone and upper lias, in Switzerland the molasse. For centuries certain wells on the continent, called Kropfbrunnen, have been known to yield a water that would cause goitre in men and animals, and they have been largely patronised by young men anxious to escape conscription. I have lately discovered a goitre well in Gloucestershire which has caused the disease, to my knowledge, in three or four of the very few persons who have drunk of its waters for any length of time.

In Khokand, Turkestan, a very large proportion of the whole population suffer, and Russian soldiers stationed there rapidly acquire the disease. Changing a water supply will sometimes induce or abate an epidemic of goitre. Thus at Rupperswyl, near Aarau, 59 per cent. of the children were goitrous. In 1884 the water supply was changed, and in ten years the percentage of goitrous children had fallen to 11.

It is a noteworthy fact that boiling the water will abolish its remarkable power. This has naturally led to a germ theory of the origin of goitre, but all attempts to find the organism have failed. It is not very probable that a germ should be distributed in particular geological horizons very widely separated from one another. A chemical theory is made much more probable by the analogy of animal experimentation. The water is still active after passing through a Berkefeldt filter. Moreover, in goitre the colloid is extraordinarily deficient in iodine, and in early cases iodides will effect a cure.

Naturally attempts have many times been made to find that the waters of goitre wells are deficient in iodine, but they have usually proved to contain a normal amount; and, indeed, this might have been foreseen. We get most of our iodine from vegetables, not from drinking water. The writer advances the tentative suggestion, shortly to be put to the proof, that the water of goitre wells contains traces of some metal which forms an insoluble, inert compound with iodine in the blood, so, as it were, stealing it from the thyroid gland. Boiling the water precipitates the metal as an insoluble carbonate. It would be mere guessing to mention names of metals, but either lead or silver, amongst many rarer, might meet the indications. If the chemical theory is correct, the water of Kropfbrunnen should cure exophthalmic goitre, in which the iodine content of the gland is excessive.

Why should the thyroid enlarge when the iodine supply is small? Manifestly to obtain a larger blood supply, to make better use of what iodine it can obtain. Similarly one kidney enlarges when the other is removed in order to capture a larger quantity of urea for excretion, and dwellers on mountain-tops increase their red blood corpuscles to make the best possible use of the attenuated supply of oxygen. It is interesting to note that both Halstead and Edmunds have proved that bitches deprived of most of their thyroids have litters of goitrous pups, and that of 2,333 cases of congenital goitre in man collected by Fabre and Thevenot the mother was almost invariably goitrous.[5] The fœtus was supplied with nutriment deficient in iodine, and became goitrous in the attempt to obtain more.

*Iodides and iodoform increase the amount of iodine in the*

*thyroid colloid, with a corresponding increase in its physiological activity.* This has been proved in man by Oswald, and in dogs by Hunt and Seidell.

Now, the secretion of the thyroid is poured, not into a duct which carries it away, but into the blood stream. When strychnine is given to a patient it is excreted by the kidney, and ceases to exert any physiological effect ; but the secretion of the thyroid does exert a physiological effect, and if over-secretion is stimulated by an excessive supply of iodiferous compounds, it is natural to expect symptoms of thyroidism.

*The constitutional symptoms of iodoform poisoning are due to acute thyroidism.* Iodoform has been recognised to cause trouble in four ways. It may give rise to—

1. Dermatitis, erythema or other rashes.
2. Toxic amblyopia (five cases) or optic atrophy (one case).
3. Haunting with the smell and taste of the drug.
4. Acute thyroid symptoms.

An overdose of thyroid extract causes rapid pulse, headache, restlessness, nausea, and delirium ; in a few cases exophthalmos has been obtained. The picture is thus very like that of Graves's disease.

I have collected about 100 cases in which iodoform is said to have caused a similar clinical picture—rapid pulse, delirium, headache, vomiting, and occasionally fever.[6] The leading pharmacologists now agree in attributing these effects to acute thyroidism. The condition is not easy to diagnose ; it is quite unlike the ordinary types of drug poisoning, and may be confounded with the symptoms of septic absorption. Probably some of the cases were not genuinely iodoform poisoning. When, however, there has been a very rapid pulse with a normal temperature, and the patient has been haunted by the smell and taste of the drug, there can be no room for doubt.

In some cases enormous quantities of the powder had been applied to wounds, one, two or even three ounces at a time. It is remarkable, seeing how frequently iodoform is used as a dressing for chronic tuberculous sinuses in children, that nearly all the recorded cases are in adults.

*In susceptible persons iodoform may light up an attack of chronic thyroidism, that is of exophthalmic goitre.* One such case has come under my notice.

**Case.**—A middle-aged lady was treated for a carbuncle in the perineum, which was dressed by a medical practitioner and a nurse with very ordinary quantities of iodoform. During three weeks about half an ounce of the powder was dusted on, and altogether about 40 inches of narrow iodoform packing gauze were used. The carbuncle healed well under this dressing. For weeks after the cessation of treatment she continually complained that she could smell and taste iodoform, though there was in reality none in the house. She went to the South of England to recoup her strength three or four weeks later, and stayed ten days. On her return she was noticeably ill; there was great emaciation, she was nearly two stone below her normal weight, the pulse was always 120 or more, the thyroid was moderately enlarged, tremor was marked, and she was exceedingly nervous and restless. There was no exophthalmos. I formed the conclusion that it was a case of Graves's disease, and Dr. Michell Clarke, who kindly saw her in consultation, made the same diagnosis. It is interesting to note that she had had a "nervous breakdown" fifteen years before, which prostrated her for two years, and in which the pulse rate was persistently quick. This may have been a similar attack.

The onset of the present illness was in January, 1909. There has been very slow but decided improvement, and now (April, 1910) she is almost well, but has to lead a very quiet life.

I connect this case with the use of iodoform because—

(*a*) The trouble followed soon after the application of the drug, although it was not diagnosed for about a month.

(*b*) She undoubtedly suffered from iodoform poisoning, because the smell haunted her long after the drug was omitted.

(*c*) Iodoform is proved to cause hyperthyroidism of the acute type, and it is therefore reasonable to suppose that in a susceptible person chronic thyroidism might be produced. It is almost universally recognised that Graves's disease is due to a chronic hypersecretion of the thyroid. The gland in this disease usually has an abnormally high iodine content.

*Practical Deductions.*—Iodoform should be used sparingly on absorbing surfaces, especially in adults. It should be avoided if there is any reason to suspect a tendency to exophthalmic goitre. The treatment of early cases of parenchymatous goitre should be directed to altering the drinking water and to introducing iodine. It would appear, since iodoform causes thyroidism more readily than iodides, that iodine ought to be given to such patients in organic combination. In the treatment of exophthalmic goitre iodine starvation should be worth a trial. This could be effected by a meat diet, and possibly by giving the water of a goitre well. It is only right to say that the writer has not had an opportunity of putting the suggestion to the test.

BIBLIOGRAPHY.

[1] RICHARDSON, *The Thyroid and Parathyroid Glands.* Philadelphia, 1905. This gives a valuable summary of our knowledge up to 1905.

[2] ALLBUTT, *System of Medicine*, vol. iv, part i, 1908. Article "Thyroid" gives a full bibliography up to 1907.

[3] HUNT and SEIDELL, "Studies on Thyroid," *Hygienic Laboratory Bulletin of Public Health*, No. 47. Washington, 1909.

[4] MARINE, *Johns Hopkins Hosp. Bull.*, 1907, xviii, 359.

[5] FABRE and THEVENOT, *Rev. de Chir.*, June 10th, 1908.

[6] See, for instance, Cutler, *Boston M. & S. J.*, 1886, cxv, 73, 101, 110. A large number of cases of iodoform poisoning are here set forth; some are not very convincing. I have found many subsequent records of other cases.

